# Vitamin D Exerts Significant Antitumor Effects by Suppressing Vasculogenic Mimicry in Breast Cancer Cells

**DOI:** 10.3389/fonc.2022.918340

**Published:** 2022-06-07

**Authors:** Khuloud Bajbouj, Abeer Al-Ali, Jasmin Shafarin, Lina Sahnoon, Ahmad Sawan, Ahmed Shehada, Walaaeldin Elkhalifa, Maha Saber-Ayad, Jibran Sualeh Muhammad, Adel B. Elmoselhi, Salman Y. Guraya, Mawieh Hamad

**Affiliations:** ^1^ College of Medicine, University of Sharjah, Sharjah, United Arab Emirates; ^2^ Sharjah Institute for Medical Research, University of Sharjah, Sharjah, United Arab Emirates; ^3^ Medical Pharmacology Department, Cairo University, Cairo, Egypt; ^4^ Department of Physiology, Michigan State University, East Lansing, MI, United States; ^5^ College of Health Sciences, University of Sharjah, Sharjah, United Arab Emirates

**Keywords:** vitamin D, breast cancer, invasion, vasculogenic mimicry, metalloproteinases

## Abstract

**Background:**

Numerous clinical and experimental observations have alluded to the substantial anti-neoplastic role of vitamin D in breast cancer (BC), primarily by inducing apoptosis and affecting metastasis. Tumor progression and resistance to chemotherapy have been linked to vasculogenic mimicry (VM), which represents the endothelial-independent formation of microvascular channels by cancer cells. However, the effect of vitamin D on VM formation in BC has not been thoroughly investigated. This study examined the impact of 1α,25-dihydroxyvitamin D3 (calcitriol), the active form of vitamin D, on the expression of major factors involved in BC migration, invasion, and VM formation.

**Experimental Methods:**

Publicly available transcriptomic datasets were used to profile the expression status of the key VM markers in vitamin D-treated BC cells. The *in silico* data were validated by examining the expression and activity of the key factors that are involved in tumor progression and MV formation in hormone-positive MCF-7 and aggressive triple‐negative MDA-MB-231 BC cells after treatment with calcitriol.

**Results and Discussions:**

The bioinformatics analysis showed that tumor VM formation-enriched pathways were differentially downregulated in vitamin D-treated cells when compared with control counterparts. Treatment of BC cells with calcitriol resulted in increased expression of tissue inhibitors of metalloproteinases (TIMPs 1 and 2) and decreased content and gelatinolytic activity of matrix metalloproteinases (MMPs 2 and 9). Furthermore, calcitriol treatment reduced the expression of several pro-MV formation regulators including vascular endothelial growth factor (VEGF), tumor growth factor (TGF-β1), and amphiregulin. Eventually, this process resulted in a profound reduction in cell migration and invasion following the treatment of BC cells with calcitriol when compared to the controls. Finally, the formation of VM was diminished in the aggressive triple‐negative MDA-MB-231 cancer cell line after calcitriol treatment.

**Conclusion:**

Our findings demonstrate that vitamin D mediates its antitumor effects in BC cells by inhibiting and curtailing their potential for VM formation.

## Introduction

Breast cancer (BC) is the most common type of cancer among women worldwide (1). Epidemiological data from 1990 to 2017 have signaled that the incidence of BC has been rising in all geographical regions of the world, especially in the Middle East and North Africa (MENA), South Asia, and Latin America (2). The staggering upsurge in the incidence of BC continues to pose a serious health challenge to the global healthcare authorities. One of the major hurdles faced by healthcare professionals remains the heterogeneous and complex nature of BC (3, 4). To add to its complexity, the breast tissue, being subject to a diverse set of conflicting hormonal and growth signals, is more prone to neoplastic transformations as opposed to other biologically less dynamic tissues.

It is well established that classical angiogenesis, initiated by endothelial blood vessels, supports tumor growth and metastasis. However, in 1991, Maniotis et al. reported an endothelial-independent vascularization formation by tumor cells, a process described as vasculogenic mimicry (VM). The VM contributes to tumor proliferation and invasion in many types of cancers through the upregulation of several proteins, including matrix metalloproteinase (MMP)-2, MMP-9, vascular endothelial growth factor (VEGF), and growth factor-β1 (TGF-β1) (5–9). Despite the technological and clinical advances in BC management and therapeutics, VM has been shown to be associated with aggressive behaviors of tumor progression and perfusions (10, 11), leading to unsatisfactory and adverse clinical outcomes (12, 13). Therefore, there is an ever-growing need for the development of VM-specific therapeutic strategies for BC.

In the last two decades, a plethora of investigational studies have explored the status of VM formation and its role in the prognosis and clinicopathological parameters of BC. For instance, a study on more than 1,200 patients with BC showed a positive correlation between the increased VM positivity and larger tumor size, the propensity for metastasis, differentiation grade, and poor prognosis (14). Considering the characteristics of breast molecular subtypes and hormone-positive BC expression of VEGF (15), research has shown that cancer progression can be arrested or slowed down by targeting TGF-β1, MMP-2, and MMP-9 when irradiated by a proton beam (7). Additionally, the ERα-positive cell line MCF-7 has been reported to induce VM upon exposure to the VM mediator, interleukin 1β (7). Furthermore, aggressive triple‐negative MDA-MB-231 BC cells readily exhibit VM phenotypes by forming tubular-like structures in the gel matrix (16).

Vitamin D is known to undergo a two-step metabolism in both the liver and kidney to produce the biologically active form calcitriol, which binds to the vitamin D receptor (VDR) and allows it to perform a variety of physiological roles (17, 18). The calcitriol, in turn, operates by binding to the intracellular VDRs in target cells. VDRs, first reported in the BC cell lines in 1979, represent a family of nuclear steroid receptors that, when engaged, can regulate the expression of greater than 200 genes involved in cell growth and differentiation and has been shown to greatly affect breast tissue kinetics (19, 20), by acting as ligand-activated transcription factors (21). Numerous extrarenal tissues in the body including breast tissue cells contain 1-α-hydroxylase enzymes needed to produce the active vitamin D metabolite 1,25(OH)2D from circulating 25(OH)D (22). Previous work has shown that the locally synthesized 1,25(OH)2D binds to VDRs expressed in breast epithelial tissue and modulates the expression of several genes (23). Breast tissue cells also contain 24-hydroxylase enzyme (CYP24) that transforms 1,25(OH)2D into the less active metabolites (24,25-dihydroxyvitamin D3 and 1,24,25-trihydroxyvitamin D3). Hence, breast tissue cells possess all key components to produce vitamin D and transduce and respond to vitamin D-dependent signals (23, 24). Numerous observational, *in vitro*, and animal model-based studies have elaborated on the protective effects of vitamin D signaling against the development and progression of BC (25–34).

The clinical administration of calcitriol or vitamin D analogs has been investigated in several epidemiological and experimental studies that have indicated its effective role in the prevention and treatment of a wide spectrum of malignancies (35, 36). Calcitriol has been shown to suppress cell proliferation and tumor progression by altering multiple mechanisms (37, 38). It inhibits cancer stem-like cells and induces triple-negative BC differentiation (39). Additionally, it has been shown that calcitriol exhibits anti-proliferative concentrations in both MCF-7 and MDA-MB-231 BC cell lines (40). We have recently reported that calcitriol could exert significant anticancer effects by disrupting cellular iron homeostasis (41). Interestingly, studies have analyzed the role of calcitriol in angiogenesis. It has been shown that calcitriol treatment would enhance angiogenesis in *in vitro* and *in vivo* lab-based experiments (42–45). In sharp contrast, the impact of calcitriol on vascularization has been shown to decrease endothelial cell growth and attenuate vessel formation (46, 47). In this perspective, a recent study has demonstrated the ability of calcitriol to inhibit tumor neovascularization and metastasis in BC (48). Collectively, more investigations are essential to investigate the antineoplastic role of vitamin D in BC. Therefore, this study was designed to investigate the anti-metastatic role of vitamin D and its association with the modulation of VM factors in BC cells.

## Materials and Methods

### Bioinformatics Analysis of Publicly Available Transcriptomic Data Resources

*In silico* bioinformatics were used to identify major pathways that are associated with vitamin D in BC cells. The microarray dataset of GSE27220 (41) was obtained from the National Centre for Biotechnology Information Gene Expression Omnibus (NCIB GEO, https://www.ncbi.nlm.nih.gov/geo). The transcriptional effect of 1,25-dihydroxyvitamin D3 was explored at physiological and supraphysiological (pharmacological) concentrations (100 nM) in the BC MCF-7 cell line. The differentially expressed genes (DEGs) were identified using the GEO2R online tool (https://www.ncbi.nlm.nih.gov/geo/info/geo2r.html), which employs LIMMA (Linear Models for MicroArray data) and GEOquery packages from the Bioconductor for group comparisons. Gene set enrichment analysis was carried out using "Enrichr" tool (49).

### Cells and Treatment Protocols

Human BRCA cell lines MCF-7 and MDA-MB-231 from the American Type Culture Collection (Manassas, VA, USA) were used throughout the study. Cells were maintained in Dulbecco’s Modified Eagle’s Medium (DMEM) supplemented with 2 μg/ml of insulin, 1 mM of sodium pyruvate, 1 mM of non-essential amino acids, 4 mM of glutamine, 10% fetal calf serum, and antibiotics (penicillin/streptomycin) at 37°C and 5% CO_2_. Cells were seeded at 0.5–1 × 10^5^ cells/ml in 25-cm flasks at ~70% confluency, and then cells were treated with various concentrations of calcitriol (25-hydroxyvitamin D; the active form of vitamin D) (2551; Tocris Bioscience, Minneapolis, MN, USA) for several time points. Control cultures were either left untreated or treated with equal volumes of dimethyl sulfoxide (DMSO) as the vehicle.

### Quantitative Real-Time Reverse Transcription–PCR

The cDNA was synthesized from 1 μg of total RNA using the QuantiTect Reverse Transcription Kit (Qiagen, Valencia, CA, USA), according to the manufacturer’s protocol. RT-PCR was performed using 1:l of complementary DNA (cDNA), specific primers for various tissue inhibitors of metalloproteinases (TIMPs) [TIMP1-forward: 5′-CGCAGCGAGGAGGTTTCTCAT-3′, TIMP1-reverse: 5′-GGCAGTGATGTGCAAATTTCC-3′, TIMP2-forward: 5′-GGCGTTTTGCAATGCAGATGTAG-3′, TIMP2-reverse: 5′-CACAGGAGCCGTCACTTCTCTTG-3′], SYBR^®^ Green I, and an iCycler Thermal Cycler. Expression levels of target human genes were normalized to GAPDH expression [GAPDH forward-5′-ATCACCATCTTCCAGGAGCGAGATC-3′, GAPDH reverse-5′-GGCAGAGATGATGACCCTTTTGGC-3′].

### Western Blotting Analysis

Cells were lysed in ice-cold NP-40 lysis buffer (1.0% NP-40, 150 mM of NaCl, and 50 mM of Tris-Cl, pH 8.0) containing protease cocktail inhibitor tablets (Cat. No. S8830; Sigma, Darmstadt, Germany). Whole-cell lysate protein concentrations were quantified using the standard Bradford method. Lysate proteins were separated by 12% sodium dodecyl sulfate–polyacrylamide gel electrophoresis (SDS-PAGE) and then transferred onto a nitrocellulose membrane. The membrane was blocked by 5% skimmed milk powder dissolved for 1 h at room temperature, washed with TBST, and reacted with primary immunoglobulin G (IgG) unlabeled antibodies (Pro-VM formation Sampler Kit, Cell Signaling Technology, Danvers, MA, USA) at 1:1,000 dilution overnight at 4°C. The secondary (anti-mouse and anti-rabbit) antibodies (Cat. No. 7076 and 7074) were then reacted with the membrane at 1:1,000 dilutions for 1 h at room temperature. The secondary (anti-IgG) antibody (Cell Signaling) was reacted with the membrane at 1:5,000 dilution for 1 h at room temperature. Chemiluminescence was detected using the enhanced chemiluminescence (ECL) kit (Cat. No. #1705061; Bio-Rad Laboratories, Hercules, CA, USA). Later, the protein band quantification was carried out using the Bio-Rad Image Lab software (ChemiDoc™ Touch Gel and Western Blot Imaging System; Bio-Rad). Then β-actin (Sigma) was used as a normalization control, and values of control (untreated) samples were defined as 1.00; values of experimental samples were quantified relative to those of control.

### Matrix Metalloproteinase Activity Assay

Cells treated with and without calcitriol were assayed for MMP activity using the human MMP-2 (Cat. No. ab100606, Abcam, Cambridge, UK) and MMP-9 assay kits (Cat. No. ab100610, Abcam, Cambridge, UK); supernatants of calcitriol-treated and control cells were separately collected at 24 and 48 h posttreatment. As per the manufacturer’s protocol, 10 µg/ml of trypsin was added and incubated for 1.5 h. A trypsin inhibitor was then added at 100 µg/ml concentration for 15 min. MMP substrate solution with test components was then added to the microplate along with the controls. Plates were read at room temperature and 412-nm wavelength absorbance, and data were tabulated and analyzed.

### Proteome Profiler Array

Fifty-five angiogenesis-related proteins were measured in MCF-7 and MDA-MB-231 cells using the Human Angiogenesis (Pro-VM formation mediators) Array Kit (Cat. No. ARY007; R&D Systems, Minneapolis, MN, USA). Whole-cell lysate protein concentrations were quantified using the standard Bradford method. Four nitrocellulose membranes, each containing 55 different capture antibodies, were blocked by Array Buffer 6 for 1 h at room temperature. Lysate aliquots containing 300 μg of protein were prepared with Array Buffer 4 and 20 μl of Detection Antibody Cocktail. Samples were then loaded onto the membrane overnight at 2°C–8°C. Chemiluminescence was detected by streptavidin–horseradish peroxidase (HRP) methods using the dilution factor suggested by the manufacturer. Protein dot quantification was done using the Bio-Rad Image Lab software (ChemiDoc™ Touch Gel and Western Blot Imaging System; Bio-Rad). Reference spots were used as a normalization control; values of control (untreated) samples were defined as 1.00; values of experimental samples were quantified relative to that of control.

### Cell Migration Assay

Cells treated with and without calcitriol were assayed using Cell Migration/Chemotaxis Assay Kit (96-well, Abcam) to measure the migration level according to the manufacturer’s instructions. Cell Migration/Chemotaxis Assay Kit (96-well, Abcam) utilizes a Boyden chamber, where the cells migrate through a semi-permeable membrane under different stimuli. Cell migration was analyzed directly by reading fluorescence (Ex/Em = 530/590 nm) in a plate reader. Prior to the assay, cells were prepared by starving the cells for 18–24 h in serum-free media. A cell migration assay containing the desired chemoattractant was prepared in the bottom chamber. The cell migration chamber was incubated at 37°C in a CO_2_ incubator for 2–48 h. The standard curve for each cell type was prepared. The migrated cells were separated. The cell dye was added, and the migrated cells were quantified.

### Cell Invasion Assay

Calcitriol-treated and control cells were assayed using the Cell Invasion Assay kit (Basement Membrane, 96-well, ab235697, Abcam, USA) to measure the invasion level according to the manufacturer’s instructions. Cell Invasion Assay (Basement Membrane, 96-well, Abcam) utilizes a Boyden chamber coated with Basement Membrane Extract (BME), where the cells invade the matrix and then migrate through a semi-permeable membrane in the Boyden chamber in response to stimulants or inhibitory compounds. Cell invasion was analyzed directly by reading fluorescence (Ex/Em = 530/590 nm) in a plate reader. Prior to the assay, cells were prepared by starving the cells for 18–24 h in serum-free media. A cell invasion assay containing the desired chemoattractant was prepared in the bottom chamber. The cell invasion chamber was incubated at 37°C in a CO_2_ incubator for 2–48 h. The standard curve for each cell type was prepared. Later, the cells were washed. The cell dye was added, and then cells were incubated at 37°C in a CO_2_ incubator for 60 min. The cell invasion chamber was disassembled, and the invading cells were quantified.

### Tube Formation Assay

Cells were seeded in 96-well plates at a density of 2 × 10 cells per well in the matrix solution and then processed according to the manufacturer’s protocol (Abcam, UK). Several images were captured by a phase-contrast inverted microscope at ×10 magnification.

### Statistical Analysis

Data were statistically analyzed by the two-way ANOVA with Tukey’s multiple comparisons test for multiple comparisons of values; a *p* < 0.05 was considered statistically significant. Data fitting and pictorial graphs were presented using the GraphPad Prism 8 software (San Diego, CA, USA).

## Results

### Vitamin D Signaling Downregulates “TGF-β Regulation of Extracellular Matrix” and “Vasculogenic Mimicry-Related” Pathways in BC Cells

**Figure 1:**
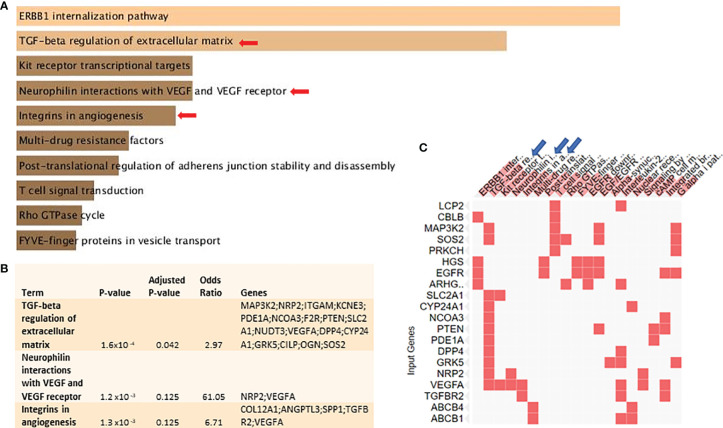
Vitamin D signaling differentially regulates key VM-related genes and signaling pathways in BC cells: with the use of Enrichr tool, a publicly available dataset of MCF-7 cells treated with pharmacological doses of 1,25-dihydroxycholecalciferol (vitamin D3) (GSE27220) was used to identify differentially regulated genes and pathways. MCF-7 cells used in generating these data were left untreated or treated with 100 nM of 1,25-dihydroxycholecalciferol (calcitriol) (treated, n = 5; control, n = 5). **(A)** Top differentially downregulated pathways. **(B)** Pathways selected for biological validation. **(C)** Input genes plotted versus enriched terms. VM, vasculogenic mimicry; BC, breast cancer.

Bioinformatics analysis using a publicly available dataset of MCF-7 cells treated with calcitriol showed that several pathways were subject to differential regulation by vitamin D signaling (**Figure 1A**). “TGF-β regulation of extracellular matrix” and “VM-related” pathways are shown in **Figures 1B, C** as the top downregulated pathways. They were also selected for biological validation. The signaling pathways, which are upregulated in calcitriol-treated MCF-7 cells, relative to the controls are shown in **Supplementary Table 1**. These data show the adjusted p-value, odds ratio, and the combined score for each pathway. It also shows the DEG related to each pathway.

### Vitamin D Influences the Levels and Activity of Tissue Inhibitors of Metalloproteinases and Matrix Metalloproteinases

**Figure 2:**
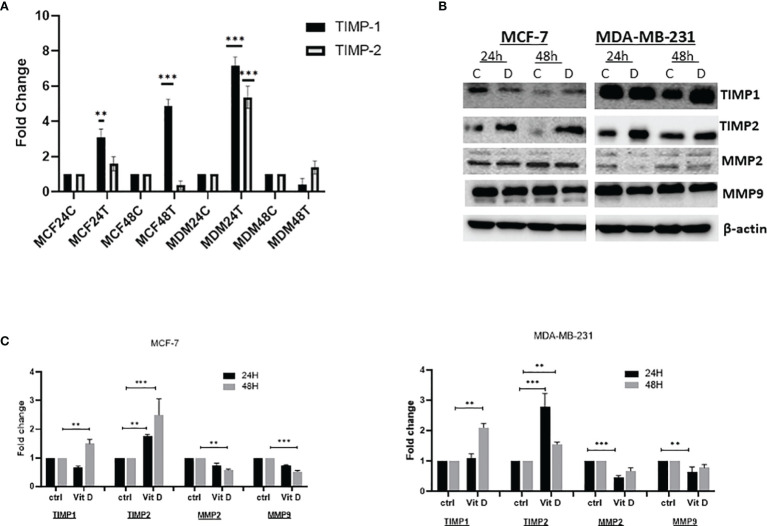
Expression levels of TIMPs and MMPs in MCF-7 and MDA-MB-231 cell lines treated with calcitriol for 24 and 48 h. **(A)** qRT-PCR analysis of TIMP1 and TIMP2 gene expression levels. **(B)** Western bolt analysis showing TIMP1, TIMP2, MMP-2, and MMP-9 protein levels in MCF-7 and MDA-MB-231 cells treated with 10 µM of calcitriol and cultured for 24 and 48 h. **(B)** ***p < 0.01, determined using unpaired two-tailed Student’s t-test. Representative immunoblots depicting protein levels where β-actin was used as loading control. **(C)** Quantitative analysis of relative protein band density after normalization to β-actin and compared in MCF-7 and MDA-MB-231 cells treated with 10 µM of calcitriol and cultured for 24 and 48 h and compared to the control. (*) represents statistically significant change in viability between the indicated treatment groups at given time points. **p < 0.05; ***p < 0.001. TIMPs, tissue inhibitors of metalloproteinases; MMPs, matrix metalloproteinases.

The effect of vitamin D was measured by analyzing the level of TIMPs and MMPs on MCF-7 and MDA-MB-231 cell lines treated with 10 µM of calcitriol for 24 and 48 h. As demonstrated in **Figure 2A**, the expression level of TIMP1 increased at both 24 and 48 h in MCF-7-treated cells compared with the control. Moreover, expression of TIMP2 also increased 24 h posttreatment, while a reduction was observed at 48 h compared to the control in MCF-7 cells. However, expression levels of both TIMP1 and TIMP2 showed a significant upregulation in MDA-MB-231 posttreatment at 24 h. Reduction of TIMP1 and non-significant TIMP2 upregulation was observed 48 h posttreatment. The treatment of MCF-7 and MDA-MB-231 cells with 10 µM of vitamin D showed a significant effect on the expression of TIMPs and MMPs at protein levels. As shown in **Figure 2B**, TIMP1 levels were significantly decreased in MCF-7 at 24 h, while an increase was observed at 48 h posttreatment. However, TIMP2 expression was increased at both 24 and 48 h. Furthermore, the expression levels of both MMP-2 and MMP-9 proteins were decreased in MCF-7 at 24 and 48 h posttreatment. However, the expression levels of both TIMP1 and TIMP2 were significantly increased in MDA-MB-231 at both 24 and 48 h post vitamin D treatment, whereas a decrease of MMP-2 and MMP-9 at both time points posttreatment was observed.

### Vitamin D Reduces Activities of MMP-2 and MMP-9

**Figure 3:**
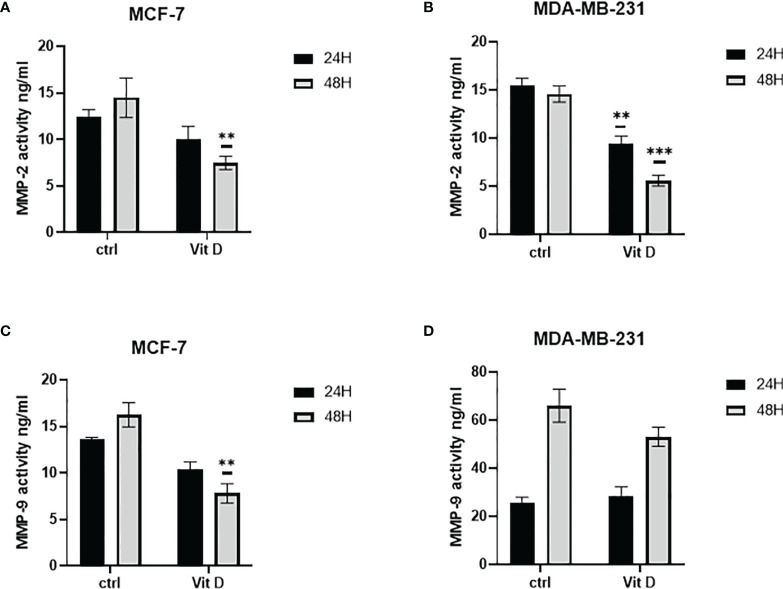
Quantitative analysis using ELISA to measure MMP-2 and MMP-9 activities. Enzymatic activity was measured using ELISA in MCF-7 **(A, C)** and MDA-MB-231 **(B, D)** cells following 10 µM of calcitriol treatment. (** and ***) represents statistically significant change (p < 0.05 and p < 0.001) in MMP-2 and MMP-9 enzymatic activity between treated and control (Ctrl) untreated cells at given time points.

The effect of vitamin D on MMP-2 and MMP-9 activity levels is illustrated in **Figure 3**. As illustrated in **Figure 3A**, the activity levels of MMP-2 in MCF-7 were decreased at both 24- and 48-h posttreatment. At the same time, the activity levels of MMP-9 were reduced at both time points after treatment (**Figure 3C**). However, the activity significantly decreased in MDA-MB-231 cells at both 24 and 48 h as compared with the control (**Figure 3B**). Additionally, MMP-9 activity increased in MDA-MB-231 treated cells at 24 h, while the activity level decreased at 48 h post calcitriol treatment (**Figure 3D**).

### Vitamin D Disrupts the Activity of Pro-Vasculogenic Mimicry Regulators

**Figure 4:**
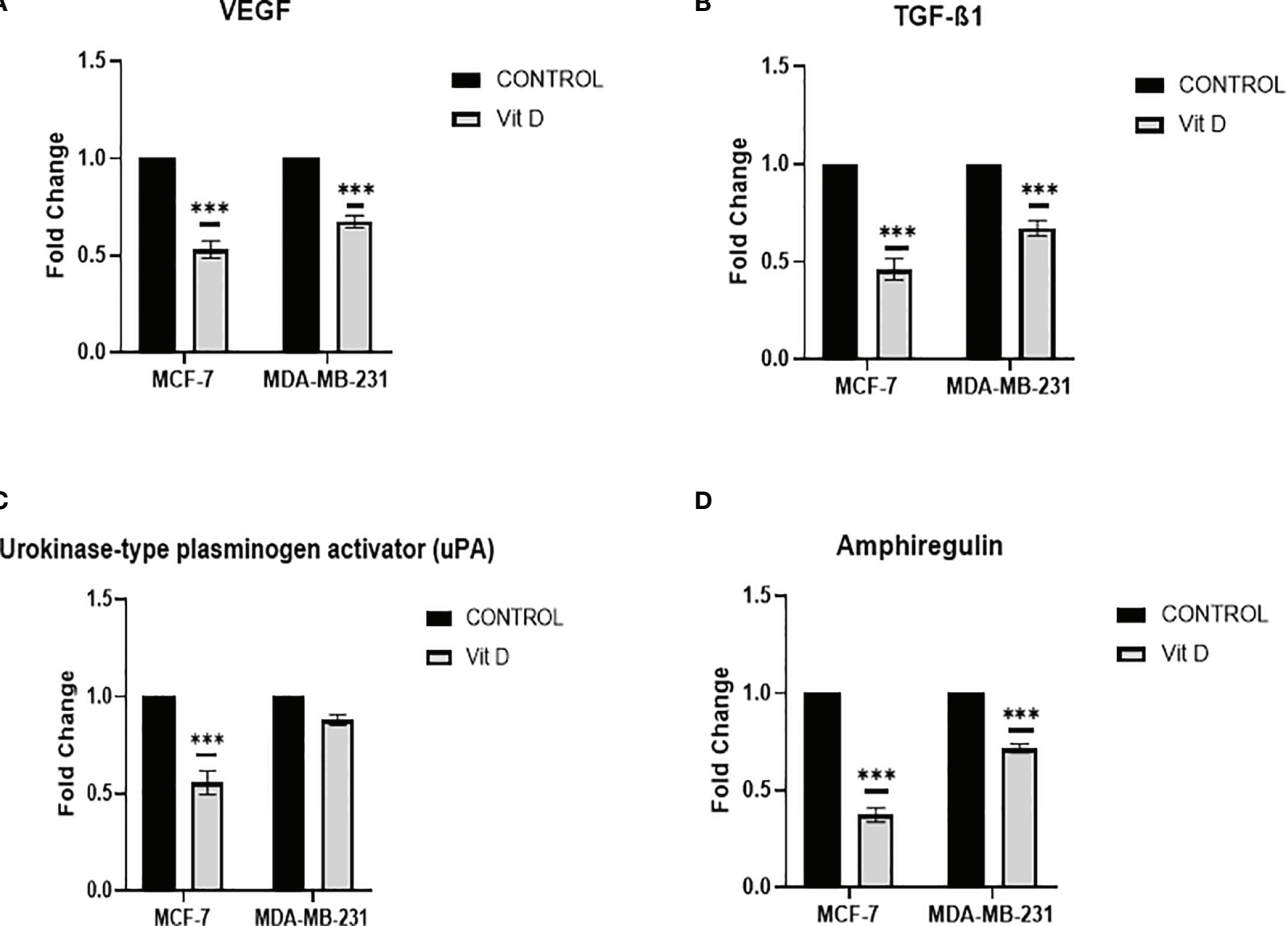
VM Proteome Profiler array analysis in MCF-7 and MDA-MB-231 cells after calcitriol treatment. VEGF **(A)** and TGF-β1 **(B)**, urokinase-type plasminogen activator (uPA) **(C)**, and amphiregulin **(D)** protein levels in MCF-7 and MDA-MB-231 cells following calcitriol treatment. (***p < 0.001) represents statistically significant change in protein levels between treated and control (Ctrl) untreated cells at given time points.

Intending to evaluate the effect of vitamin D on VM mechanism, we investigated the regulators of pro-VM, by Proteome Profiler array analysis, which affirmed that vitamin D treatment has remarkably reduced the levels of fundamental pro-VM regulators in MCF-7 and MDA-MB-231 cells. A significant reduction of VEGF was observed in MCF-7 and MDA-MB-231 cells posttreatment (**Figure 4A**). Additionally, the TGF-β1 level was also significantly reduced in both cell lines compared to the control (**Figure 4B**). Moreover, there was a significant reduction of urokinase-type plasminogen activator (uPA) level that was more consequential in MCF-7 cells in distinction to MDA-MB-231 cells (**Figure 4C**), as the level of amphiregulin decreased in both MCF-7 cells and MDA-MB-231 cells, but more significant reduction was observed in MCF-7 after treatment as compared to the control (**Figure 4D**). The panel of all VM regulating proteins is shown in **Supplementary Figure 1**.

### The Impact of Vitamin D on Cell Migration and Invasion

**Figure 5:**
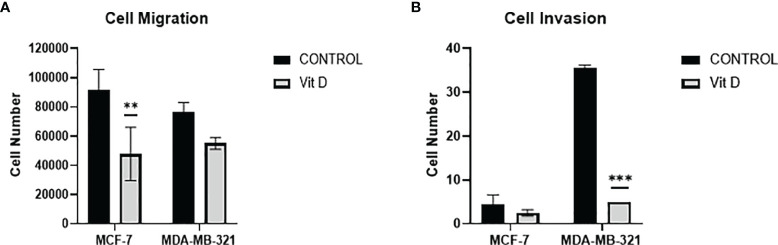
Migration and invasion of MCF-7 and MDA-MB-231 cells after calcitriol treatment. **(A)** Cell migration assay displays the reduction of the number of migrating cells in both MCF-7 and MDA-MB-231 cells after treatment with calcitriol (10 µM). Cell invasion assay displays the reduction of the number of migrating cells in both MCF-7 and MDA-MB-231 cells after treatment with calcitriol (10 µM). (**), (***) represents statistically significant change (*p* < 0.05) and (*p* < 0.001), respectively in number of cells between treated and control (Ctrl) untreated cells at given time points.

To discern the impact of vitamin D on the dynamics of cell migration and cell invasion, we quantified the migration/invasion of untreated and treated MCF-7 and MDA-MB-231 cells (**Figures 5A, B**). Both cell lines MCF-7 and MDA-MB-231 displayed a significant reduction in the migration level after vitamin D treatment (**Figure 5A**). Furthermore, calcitriol treatment inhibited the invasion ability of both cell lines, with the MDA-MB-231 cell line displaying a significant reduction in the number of invading cells (**Figure 5B**).

### The Effect of Vitamin D on Vasculogenic Mimicry Formation

**Figure 6:**
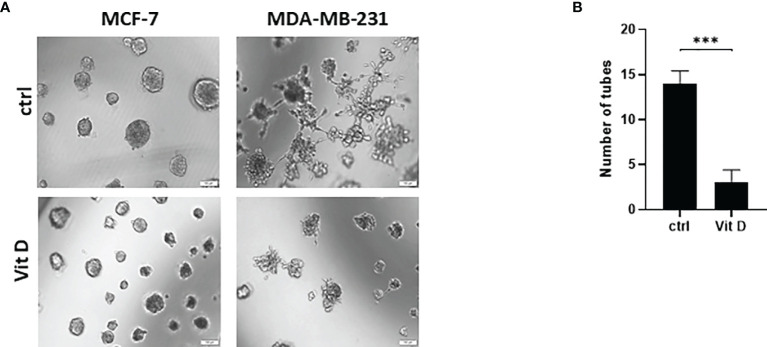
The effect of calcitriol treatment on tube formation assay in MCF-7 and MDA-MB-231 cells after calcitriol treatment. **(A)** Reduction of the cell mass of both MCF-7 and MDA-MB-231 cells after treatment with calcitriol (10 µM). **(B)** Significant inhibition of tube formation in MDA-MB-231 cells after treatment with calcitriol. (***) represents statistically significant change (*p* < 0.001) in number of cells between treated and control (Ctrl) untreated cells at given time points.

We next performed the tubular-structure initiation assay, as an established *in vitro* assay for VM formation, in the MDA-MB-231 and MCF-7 cells in the control and treated groups (**Figure 6**). The Matrigel-based assay was performed to acquire the evidence of VM by analyzing the tube formation in the seed cells. This showed a reduced number and mass of MDA-MB-231 and MCF-7 cells when treated with calcitriol (**Figure 6A**). Furthermore, the tubular-structure formation was significantly decreased in the aggressive triple-negative MDA-MB-231 cells in comparison to the control counterparts (**Figure 6B**).

## Discussion

VM is a novel tumor vascular model that explicitly underpins the ability of aggressive cancer cells to form vessel-like networks that supply sufficient blood supply for tumor growth. VM induction is mediated by several molecular mechanisms and signaling pathways. Cancer stem cells (CSCs) and epithelial–mesenchymal transitions have also been linked to VM formation. VM is associated with tumor invasion, metastasis, and poor oncological outcomes. Because of the importance of VM in tumor progression, more VM-related anticancer strategies are being adopted in the medical field. Our study illustrates the VM properties of vitamin D in BC cells as vitamin D treatment induced TIMP1 and TIMP2 expression levels and reduced MMP-2 and MMP-9 catalytic activities. Similarly, the VEGF and TGF-β1 protein contents were significantly downregulated in both groups of BC cells. Overall, the migration and invasion potential were substantially downregulated by vitamin D treatment in BC cells. In addition, vitamin D reduced the cell mass and VM formation in both groups of BC cells. Finally, MMPs are essential for tumor invasion, metastasis, and VM formation.

A fundamental prerequisite for VM formation is the expression of high levels of MMPs. MMPs cleave Laminin5γ2 into 5γ2x and 5γ2′ for dense extracellular matrix protein deposition, resulting in the formation of *de novo* blood vessels in solid tumors (50). Type IV collagens are the primary building blocks of the extracellular matrix and basement membrane. Tumor cells can primarily express MMP-2 and MMP-9 to debase type IV collagens and disrupt these tissue barriers, which stimulates tumor cell invasion and metastasis (51). TIMPs inhibit MMP activity, which is required for extracellular matrix turnover in both physiologic and pathologic tissue remodeling. In addition to inhibiting MMP, they are associated with other biological systems needed for metastasis and VM (52). In this context, our study has investigated the non-neoplastic functions of vitamin D on TIMP/MMP systems that stimulate cell invasion and migration in BC. Our study findings report that MCF-7 and MDA-MB-231 cells treated with calcitriol (10 µM) resulted in increased levels of TIMP1 and TIMP2 that were most apparent after treatment for 24 h. In contrast, MCF-7 and MDA-MB-231 cells treated with calcitriol (10 µM) resulted in decreased levels of MMP-2 and MMP-9. The VEGF signaling is a key modulator of VM (53). In ovarian cancer, VEGF-A has been linked to VM formation by elevating the expression of MMP-9, MMP-2, VE-cadherin, and EphA2. The VEGFR-2 is abundantly expressed in vascular ECs, resulting in vasculogenesis. VEGFR-1, on the other hand, is overexpressed in VM-forming tumor cells in malignant melanoma (54). The elevated levels of VEGF and VEGFR-1, as well as MMP-9 and MMP-2, have been linked to the formation of VM in gastric cancer tissues (55). VEGF signaling further activates the PI3K/PKC and ERK signaling pathways, resulting in cell migration, invasion, and proliferation (56, 57). In breast and pancreatic cancer, inhibiting EphA2 reduces VEGF expression with the resultant angiogenesis *in vivo*. This finding lends credence to the theory that VEGF signaling is the activating event in VM formation (58, 59). Increased VEGFR-2 expression has been correlated with VM formation in tumors derived from CSCs and glioma stem-like cells (60, 61). A recent study demonstrated that siRNA-based VEGF gene silencing reduced cell migration, invasion, and proliferation in choroidal melanoma. VEGF inhibition reduced the expression of MMPs, AKT, p-AKT, MMP-9, and MMP-2, and thus the formation of VM was reduced through the PI3K/AKT signaling pathway (62).

Research has shown that TGF-β regulates cell cycle, cell proliferation, motility, invasion, and apoptosis (63). TGF-β can either stimulate or inhibit cancer progression in a variety of cancers. Endoglin (CD105), a TGF-β co-receptor, has been shown to induce VM formation and neo-angiogenesis in Ewing’s sarcoma (64). In a study, TGF-β was inactivated by silencing TGF-R1, with the associated reduction in the expression of MMP-2, VE-cadherin. In glioma, inhibiting the TGF-β signaling pathway reduces the expression of MMP-14 and MT1-MMP, leading to a significant decrement in the formation of VM (65, 66). Previous studies have established the role of VEGF (67), TGF-β1 (61), uPA (68), and amphiregulin (69) in VM formation in cancer. Vitamin D has anti-VM ramifications by decreasing the expression of VM growth factors in tumor cells’ VEGF (70). Consequently, in our study, we have elucidated the anti-VM potential of vitamin D in BC by reducing the level of VEGF. This finding is grounded by a reduction of fundamental pro-VM regulators VEGF, TGF-β1, and uPA, in addition to amphiregulin in MCF-7 and MDA-MB-231 cells treated with calcitriol (10 µM).

In patients with malignant tumors, VM is significantly correlated with elevated tumor grade, invasion, metastasis, and a poor prognosis (71, 72). VM emerges in a wide range of cancer tissues including aggressive melanomas (73), breast cancer (74), ovarian cancer (75), prostate cancer (76), lung cancer (77), liver cancer (78), and glioblastoma (79). The tumors with a high degree of overall VM showcase poor prognosis (80), as VM also correlates with tumor staging (81). Tumor cells that engage in VM exhibit elevated cancer stemness and endothelial-like gene expression. Tumor cells are directly adjacent to blood flow during the development of vascular mimetic vessels, increasing the likelihood of detachment and intrastation of these cells to distant sites (82). In our study, we evaluated the levels of cell migration and invasion following the vitamin D treatment of both cell lines MCF-7 and MDA-MB-231. The results depicted a clear reduction of migrating and invading cells. Human BC tumors are essentially categorized according to the clinicopathological and histopathologic characteristics along with their molecular markers. TNBC and HER2 are widely regarded as the most aggressive phenotypes of BC. The relationship between VM and breast tumor phenotype has been widely studied. *In vitro* studies have revealed that TNBC aggressive cells, as opposed to more differentiated BC cells, are more susceptible to forming tubular structures (83). Several studies have reported that TNBC MDA-MB-231 and HCC1937 cells readily form tubular-like structures (11, 84). In contrast, the ER-positive cell line MCF-7 has been shown to be incapable of forming VM (16), but in the availability of VM drivers such as interleukin 1, MCF-7 cells formed microvessel-like intersections and cords (7). Accordingly, we further investigated the effect of vitamin D on VM formation in the vitamin D-treated MDA-MB-231 and MCF-7 cells. Both cell lines exhibited a reduction in the cell mass; in addition, the tubular-structure formation was substantially reduced in the aggressive MDA-MB-231 cells. VM triggers tumor growth, progression, metastasis, invasion, and treatment failure. Numerous studies (85–87) reported that patients with VM-positive tumors have a worse prognosis and a poor 5-year survival rate than patients with VM-negative tumors. The prevalence of VM positivity, as well as its influence on clinicopathological parameters and prognosis in BC patients, has been extensively researched over the last two decades (88–90). The current body of literature affirms a negative correlation between VM and the reported clinical oncological outcomes. There is now concrete evidence that the formation of VM is a significant impediment to anti-angiogenic therapy. Admittedly, inducing hypoxia may endorse VM, which in turn promotes distant metastasis (91, 92). In a study of triple-negative BC cells, the influence of anti-angiogenic treatment on VM promotion was confirmed (93). Thus, cells treated with sunitinib (a VEGFR tyrosine kinase inhibitor) showed an increase in VM-positive cases when compared to control cells. Overexpression of HIF-1, VE-cadherin, and Twist1 was found to be responsible for these effects (93). A recent study used trastuzumab, a drug that engages the receptor tyrosine kinase HER2 in BC cells (94). Numerous VM markers were highly expressed in trastuzumab-treated cells, indicating that trastuzumab-resistant HER-2-positive BC cells can exhibit VM in an angiogenic microenvironment. As a result, VM may be recognized as one of the major causative factors of resistance to anti-angiogenic therapy in solid tumors. Conclusively, our study established a novel role of vitamin D in suppressing VM in BC cells.

## Conclusion

Our study provides compelling evidence that the antitumor and anti-VM roles of vitamin D is mediated by reducing the VM growth factor levels and by altering TIMP/MMP systems in BC. These antitumor effects of vitamin D ultimately have the potential to reduce the risk of tumor cell migration and invasion. Moreover, our study findings provide a translational significance of utilizing vitamin D (25-hydroxyvitamin D (25(OH)D) or calcitriol) as a supplementary anticancer agent.

## Data Availability Statement

The original contributions presented in the study are included in the article/**Supplementary Material**. Further inquiries can be directed to the corresponding author.

## Author Contributions

Conceptualization: KB. Data curation: KB, AA-A, LS, MS-A, and ASa. Investigation: KB, AA-A, MS-A, LS, JS, ASa, Ash, WE, JM, SG, and MH. Supervision: KB. Writing—original draft: KB, AAA, MS-A, and LS. Writing—review and editing: KB, AE, and SG. All authors agree to be accountable for all aspects of the work. 

## Funding

This work was supported by the University of Sharjah Seed grant, Ref. number: 1901090150.

## Conflict of Interest

The authors declare that the research was conducted in the absence of any commercial or financial relationships that could be construed as a potential conflict of interest.

## Publisher’s Note

All claims expressed in this article are solely those of the authors and do not necessarily represent those of their affiliated organizations, or those of the publisher, the editors and the reviewers. Any product that may be evaluated in this article, or claim that may be made by its manufacturer, is not guaranteed or endorsed by the publisher.

## Supplementary Material

Supplementary Figure 1 Images of the nitrocellulose proteome profiler membrane showing differences in the protein expression of Pro-VM formation mediators in the control and treated samples, **(A)** MCF-7 and **(B)** MDA-MB-231.

Supplementary Table 1 Key VM-related genes and signaling pathways in Breast cancer cells that are differentially regulated by Vitamin D signaling. The adjusted p-value, odds ratio, and the combined score are shown for each pathway.
