# Supplementary figures and images for: Vitamin D Exerts Significant Antitumor Effects by Suppressing Vasculogenic Mimicry in Breast Cancer Cells
Source: Front Oncol. 2022 Jun 7;12:918340. doi: 10.3389/fonc.2022.918340 (PMC9210804; doi:10.3389/fonc.2022.918340)

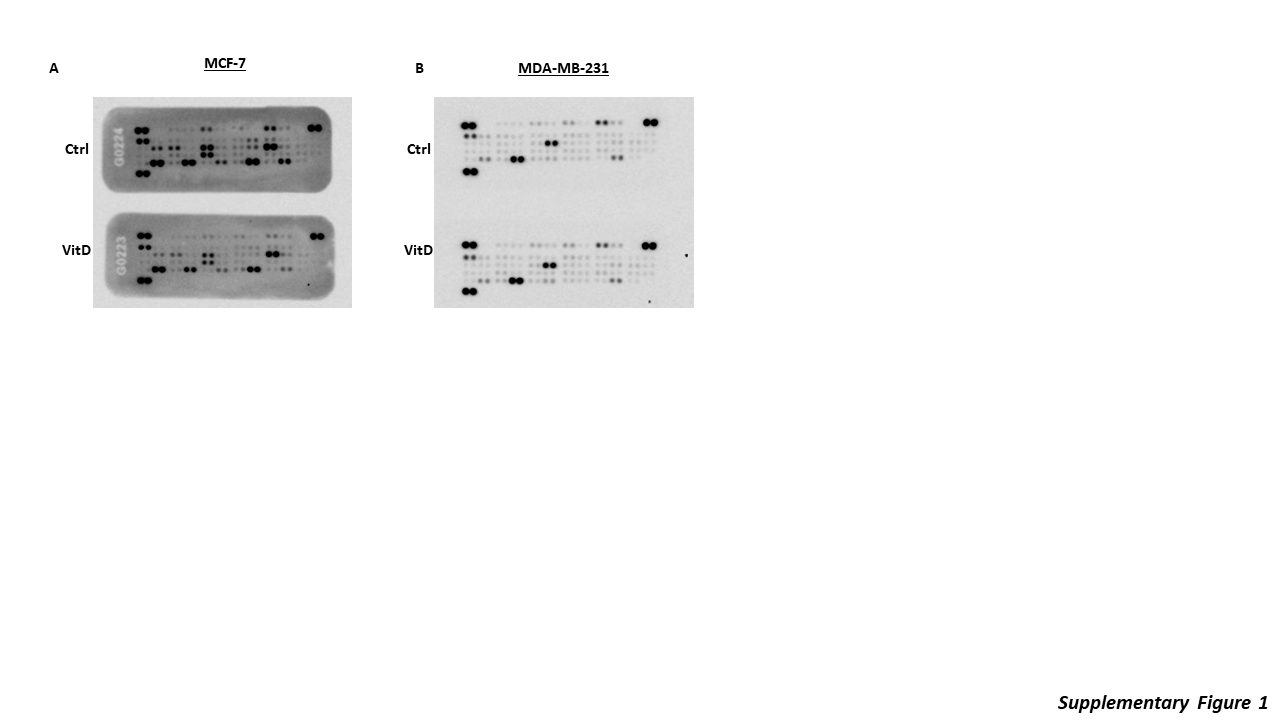

Supplement: Supplementary Figure 1 — Images of the nitrocellulose proteome profiler membrane showing differences in the protein expression of Pro-VM formation mediators in the control and treated samples, (A) MCF-7 and (B) MDA-MB-231. [file Image_1.tif]
